# *In situ* Engineering of Hollow Porous Mo_*2*_C@C Nanoballs Derived From Giant Mo-Polydopamine Clusters as Highly Efficient Electrocatalysts for Hydrogen Evolution

**DOI:** 10.3389/fchem.2020.00170

**Published:** 2020-04-07

**Authors:** Suli Liu, Xueqin Mu, Ruilin Cheng, Shiyu Lin, Yang Zhu, Changyun Chen, Shichun Mu

**Affiliations:** ^1^Department of Chemistry, Nanjing Xiaozhuang University, Nanjing, China; ^2^State Key Laboratory of Advanced Technology for Materials Synthesis and Processing, Wuhan University of Technology, Wuhan, China

**Keywords:** molybdenum carbide, mo-polydopamine, porous nanostructures, electrocatalyst, hydrogen evolution reaction

## Abstract

Low-cost and highly effective catalysts are crucial to the electrocatalytic hydrogen evolution reaction (HER). Among non-noble catalysts, molybdenum carbides are promising candidates because of their high reserves, stability, low cost, and structural diversity. In this work, we report a simple method to fabricate a hollow porous Mo_2_C@C nanoball through a hydrothermal preparation process of molybdenum precursors at high temperatures. Specifically, we have combined interfacial polymerization and the chelation effect to synthesize the Mo-polydopamine (Mo-PDA) precursor. As a result, Mo_2_C@C-3 only requires an ultralow Tafel slope (~55 mV dec^−1^) and low overpotential (η_50_ ≈ 167 mV) in a 0.5 M H_2_SO_4_ solution with long-term cycling stability. Besides, it also exhibits outstanding activity and stability under extensive HER testing in alkaline media. This study is promising for the development of advanced molybdenum carbide electrocatalysts toward electrochemical applications.

## Introduction

Hydrogen has been considered as a renewable, clean, and green energy source alternative to carbon-based fossil fuels for satisfying the ever-growing energy demand of the future (Huang C. et al., 2019; Tao et al., 2019; Takahashi et al., 2020). The hydrogen evolution reaction (HER) process is regarded as a best available strategy for producing high-purity hydrogen from abundant water (Huang C. et al., 2019; Jia et al., 2020). Platinum (Pt) and its alloys are commonly considered as benchmark catalysts for the HER, but the high cost largely impedes its commercial applications (Alinezhad et al., 2019; Park et al., 2019; Jia et al., 2020). Recently, earth-abundant catalysts, especially 3d transition metal compounds, have been reported (Li et al., 2017, 2018; Huang C. et al., 2019; Zhu et al., 2020. Among them, Molybdenum carbides (Mo_x_C) are widely used catalysts that possess a similar electronic state of having noble Pt at the Fermi level, of which noble Pt is an ideal candidate as an inexpensive metal catalyst for the HER process (Huang C. et al., 2019; Zhu et al., 2019). However, until now, most of the Mo_x_C catalysts are synthesized by the high carburization temperature method, which accelerates the agglomeration and sintering of Mo_x_C nanomaterials, inhibiting the further improvement of electrocatalytic activity (Huang H. W. et al., 2019). Therefore, to obtain satisfactory electrocatalysis activity, developing an efficient strategy to construct advanced Mo_x_C with a highly specific surface area and a variety of active sites remains a great challenge.

Nanocarbon hybridization has demonstrated impressive HER activities because it can effectively modify the electronic structure of catalysts, but their electrochemical accessibility is greatly depressed (Tang and Zhang, 2017; Wang Y. Q. et al., 2019; Zhang et al., 2020). Most importantly, during high-temperature thermal treatments, Mo_x_C particles derived from those small precursors are likely to grow into larger particles, leading to poor catalytic activity. Thus, the design of a porous architecture can provide abundant active sites, resulting in significantly improved electrochemical performances (Park et al., 2018). Although substantial progress has been made, the controllable synthesis of hollow porous Mo_x_C@C nanomaterials with high activity is still highly challenging.

**Scheme 1:**
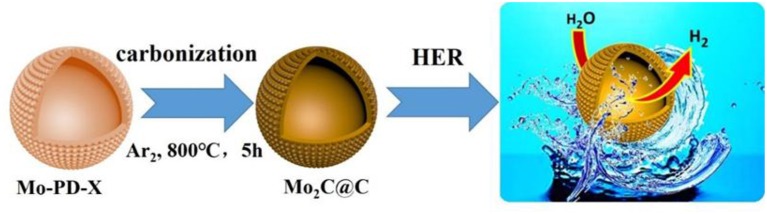
Schematic illustration for the synthesis of hollow porous Mo_2_C@C nanoball catalysts.

Herein, we initiated an *in situ* strategy to synthesize a hollow porous Mo_2_C@C nanoball anchored on carbon substrates starting with a Mo-polydopamine (Scheme 1), and then investigated their trend in electrocatalytic activity for HER at different pH values. Consequently, the hollow porous Mo_2_C@C nanoball electrode exhibited remarkable electrocatalytic activity for HER in both acidic and alkaline conditions, which are superior to those of some recently reported Mo_2_C-based electrocatalysts and close to that of commercial 20% Pt/C. A detailed investigation revealed that the incorporation of carbon significantly reduces the agglomeration and induces strong electronic interaction between Mo_2_C and C, improving the electrical conductivity and resulting in an enhanced HER performance.

## Results

As illustrated in Scheme 1, highly dispersed hollow porous Mo_2_C nanoballs anchored on carbon are obtained by a hydrothermal process of molybdenum precursors at high temperatures. Firstly, Mo reacts with dopamine hydrochloride, and different amounts of dopamine hydrochloride precursors are first mixed with an aqueous suspension of polydopamine (PDA)-coated Mo under vigorous stirring in the presence of ammonia, resulting in the precipitation of hollow porous materials. They are denoted as Mo-PD-X, where X refers to the mass of 3-hydroxytyramine in reactions. Meanwhile, the mass ratio of 1:2 for Mo: dopamine hydrochloride is the optimal condition. Then, the resulted precipitation of Mo-PD-X precursors is heated to 800°C in Ar and kept for 5 h to form Mo_2_C@C catalysts.

**Figure 1:**
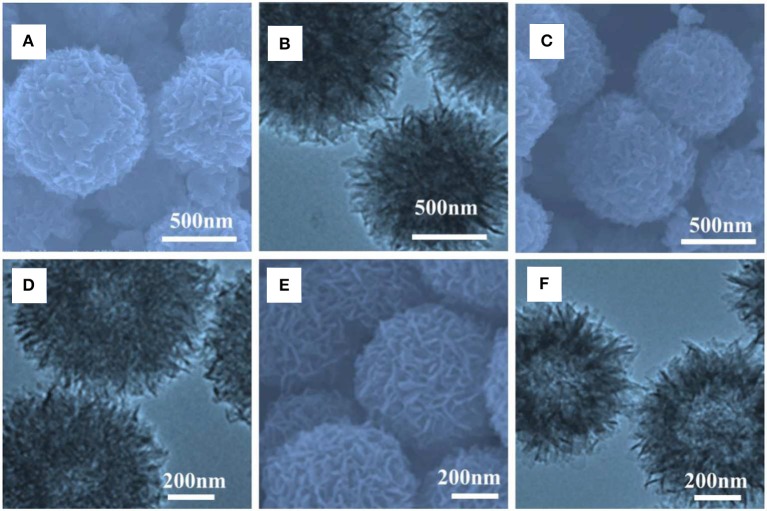
SEM and TEM images of **(A,B)** Mo-PD-1, **(C,D)** Mo-PD-2, and **(E,F)** Mo-PD-3.

First of all, the morphology of the Mo-polymelamine precursor with varied 3-hydroxytyramine hydrochloride concentration (100, 200, and 300 mg) were observed by scanning electron microscope (SEM) and transmission electron microscope (TEM), and the corresponding pyrolyzed products anchored on carbon were obtained. Figures 1A–F presents representative SEM and TEM images of Mo-PD-X at different magnifications, which exhibit a similar structure to those of the hollow porous nanoball precursors. Further TEM observation (Figures 1E,F) reveals that the Mo-PD-3 sphere possesses an ultrathin shell composed of nanosheets about several nanometers in size, among which numerous mesopores are generated. Moreover, the outer layer is dendritic, which would endow abundant low-coordinated sites on the branch surface and electron transfer pathway (Zhong et al., 2018; Chen et al., 2019; Zhang et al., 2019). In addition, the thickness of the outer layer is controllable by varying the dopamine hydrochloride precursor amount.

**Figure 2:**
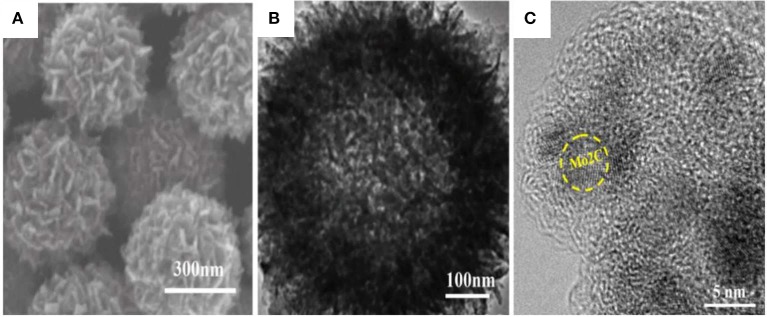
**(A)** The SEM, **(B)** TEM, and **(C)** HRTEM images of Mo_2_C@C.

The as-prepared hollow porous Mo-PD-X nanoballs were further annealed in Ar atmosphere at 800°C for 5 h to obtain hollow porous Mo_2_C@C nanoballs. In detail, the PDA inside the precursor was gradually decomposed and released from the PD-X cover. In the meantime, Mo reacted with PD-X, and then the Mo-based shell formed to self-support a hollow structure. The panoramic SEM and TEM images (Figures 2A,B) showed that the overall spherical morphology was well-preserved. Compared to the Mo-PD-X hollow nanoballs, the size of the hollow porous Mo_2_C@C nanoball shrank to ca. 500 nm. Furthermore, Figure 2C presents a high magnification TEM (HRTEM) image of Mo_2_C dendritic outer layers, and their lattice spacing (≈0.23 nm) was matched with (002) crystallographic planes of hexagonal Mo_2_C. Meanwhile, the carbon layer and porous channels of Mo_2_C/C can be clearly observed, indicating the formation of the charge-transfer pathway during the HER process. The carbon layer also can stabilize the hollow porous Mo_2_C cores to avoid deactivation and structure collapse during cyclic tests (Chen et al., 2016; Mir and Pandey, 2018; Zhu et al., 2018).

**Figure 3:**
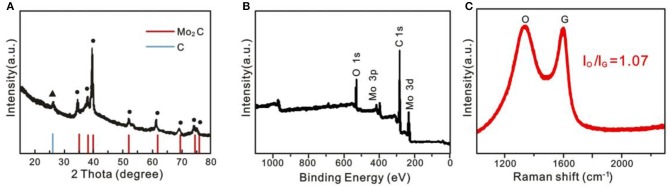
**(A)** XRD pattern, **(B)** XPS survey spectra, and **(C)** Raman spectra of hollow porous Mo_2_C@C nanoballs.

X-ray diffraction (XRD) (Figure 3A) and X-ray photoelectron spectroscopy (XPS) (Figure 3B) were also performed, and this certified a Mo_2_C/C nanostructure. From Figure 3A for Mo_2_C@C, besides the diffraction peaks from C, the other characteristic peaks are shown at 34.5, 38.0, 39.6, 52.3, 61.9, 69.8, 75.0, and 76.0°, which can only be ascribed to the hexagonal β -Mo_2_C phase (JCPDF#35-0781) (Mir and Pandey, 2018). Moreover, according to previous work, the value (I_D_/I_G_ =) of Mo_2_C@C is 1.07, implying that more unordered carbon should increase with rich defects in Mo_2_C@C, which promotes the activity (Wang Y. H. et al., 2019). Considering the TEM, XRD, XPS, and SEM results together, we can conclude that the porous Mo_2_C nanoballs were formed in the graphitic carbon.

**Figure 4:**
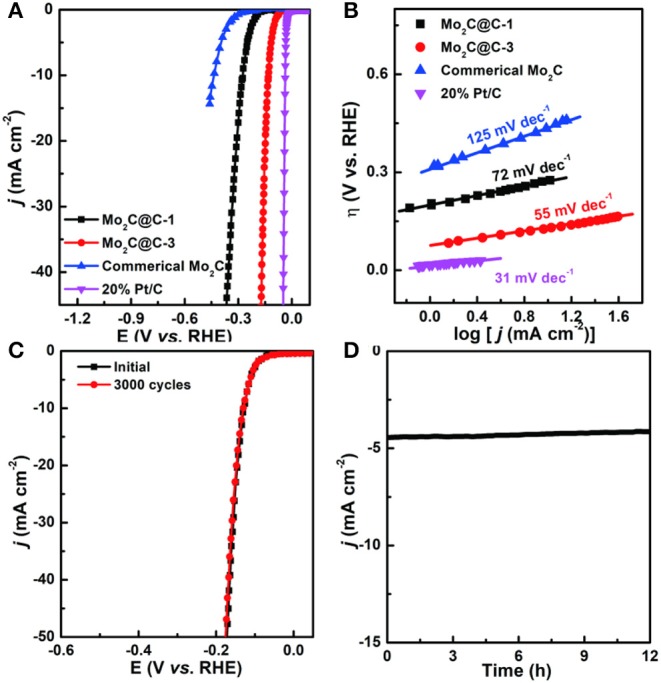
**(A)** LSV curves and **(B)** Tafel plots of Mo_2_C@C-1, Mo_2_C@C-3, 20% Pt/C, and commercial Mo_2_C in 0.5 M H_2_SO_4_ solution. **(C)** Cycling stability of Mo_2_C@C-3 before and after 3,000 cycles and **(D)** Galvanostatic data of the Mo_2_C@C-3 for 12 h in 0.5 M H_2_SO_4_ solution.

The electrocatalytic activity in the HER was evaluated by linear sweep voltammetry (LSV) in N_2_-saturated 0.5 M H_2_SO_4_ with a three-electrode electrochemical system (Chen et al., 2019; Liu et al., 2019a). For comparison, the HER activities of commercial Mo_2_C, commercial 20% Pt/C, Mo_2_C@C-1 (Mo-PD-1), and Mo_2_C@C-3 (Mo-PD-3) were also investigated, as shown in Figure 4A. Remarkably, the overpotential (η) for Mo_2_C@C-3 was small (only ≈ 129 mV) for achieving 10 mA cm^−2^, much lower than that for Mo_2_C@C-1 (271 mV) and commercial Mo_2_C (436 mV) catalysts. More significantly, the catalytic overpotential of Mo_2_C@C is lower than most non-noble metals based HER catalysts reported so far (Table S1). Meanwhile, the reaction kinetics and the rate-determining step for HER were further investigated by Tafel plots (Figure 4B and Table S1). Herein, the Tafel analysis of the Pt/C exhibited the lowest Tafel slope of 31 mV dec^−1^, suggesting that the Tafel reaction was the rate-limiting step (Xiang et al., 2018; Zheng et al., 2018). By comparison, the Tafel slope for Mo_2_C@C-3 (55 mV dec^−1^) was smaller than that for commercial Mo_2_C (125 mV dec^−1^) and Mo_2_C@C-1 (72 mV dec^−1^) catalysts, implying a faster HER rate. Additionally, the Mo_2_C@C-3 catalyst showed negligible change after 3,000 CV cycles (Figure 4C), reflecting superior durability in an acidic solution. The corresponding time-dependent potential curve (Figure 4D) further confirmed such stability. Clearly, the hollow porous Mo_2_C@C nanoball was a high performance HER catalyst in acidic solutions.

**Figure 5:**
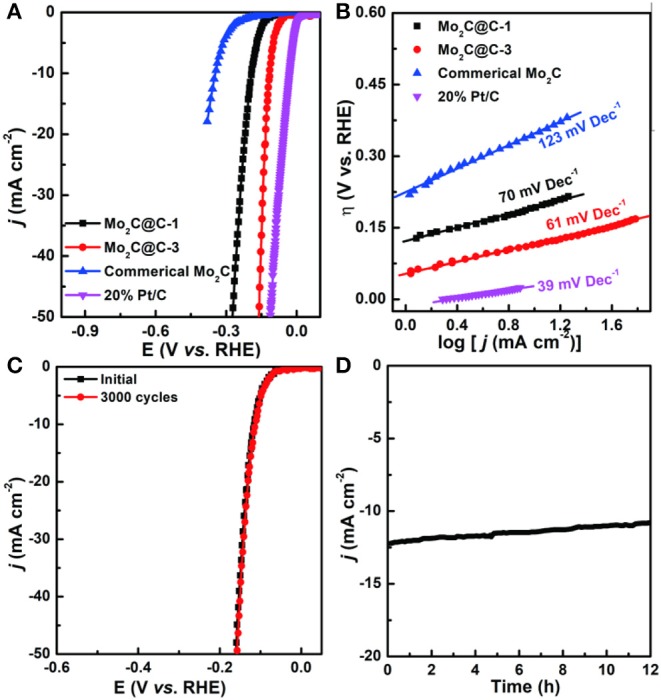
**(A)** LSV curves and Tafel plots of Mo_2_C@C-1, Mo_2_C@C-3, Pt/C, and commercial Mo_2_C in a 1.0 M KOH solution. **(B)** Tafel plots of Mo_2_C@C-1, Mo_2_C@C-3, 20% Pt/C, and commercial Mo_2_C in 1.0 M KOH solution. **(C)** LSV curves of Mo_2_C@C-3 before and after 3,000 CV cycles in a 1.0 M KOH solution. **(D)** Time-dependent current density curves of Mo_2_C@C-3 for 12 h.

To broaden the application of catalysts, the HER performance of the samples prepared above in 1.0 M KOH aqueous solutions were also then examined by electrochemical measurements (Zheng et al., 2018; Liu et al., 2019b). Figure 5A shows the polarization curves of different samples after iR correction. Interestingly, Mo_2_C@C-3 also exhibits high activity under alkaline conditions with an overpotential of 115 mV, achieving a current density of 10 mA cm^−2^, which is much smaller than those of Mo_2_C@C-1 (191 mV) and commercial Mo_2_C (347 mV). Furthermore, the HER mechanism over these catalysts was studied by corresponding Tafel plots. As shown in Figure 5B, Mo_2_C@C-3 exhibits a low Tafel slope of 61 mV dec^−1^, indicating that the HER process occurs via the Volmer–Heyrovsky mechanism, and the electrochemical desorption (Heyrovsky step) is the rate-determining step (Zheng et al., 2018). Such superior activity is significantly better than or at least comparable with those reported for Mo_x_C-based electrocatalysts (Table S2). Besides, the Mo_2_C@C-3 electrode also presented excellent durability in alkaline media (Figures 5C,D).

## Discussion

Significantly, the superior HER performance of hollow porous Mo_2_C@C nanoballs is highly correlated to the following aspects: (1) The hierarchical porous structure not only endows a high specific surface area and mitigates aggregation during carbonization process, but also facilitates efficient mass transfer of reactants and products, enhancing the HER performance (Park et al., 2018; Chen et al., 2019). (2) The presence of C not only prevents the aggregation of Mo-based compounds, but also accelerates the rate of charge transfer during HER (Chen et al., 2016; Zhu et al., 2018; Wang Y. H. et al., 2019; Wang Y. Q. et al., 2019).

In summary, hollow porous Mo_2_C nanoballs nested on carbon were fabricated by a *in situ* carbonization. The Mo_2_C@C-3 material was identified as low-cost and highly effective electrocatalysts for HER. It only needed overpotentials of 129 mV and 115 mV to drive a current density of 10 mA cm^−2^ in 0.5 M H_2_SO_4_ and 1 M KOH, respectively, and also exhibited robust catalytic stability for at least 12 h. This remarkable performance can be attributed to its unique hollow porous structure with carbon matrix. Undoubtedly, such a high-performance catalyst has promising potential to be commercialized in the future.

## Data Availability Statement

All datasets generated for this study are included in the article/Supplementary Material.

## Author Contributions

The original manuscript, figures, tables, and the Supplementary Materials were prepared by SLiu. The experimental data were prepared by XM. RC provided the original idea, helpful discussions, and the contribution in the manuscript revision. SM and CC conceived the idea. XM, RC, SLin, and YZ carried out the experiments. SLiu, XM, and RC analyzed the data.

### Conflict of Interest

The authors declare that the research was conducted in the absence of any commercial or financial relationships that could be construed as a potential conflict of interest.
