# Supplementary material for: In situ Engineering of Hollow Porous Mo2C@C Nanoballs Derived From Giant Mo-Polydopamine Clusters as Highly Efficient Electrocatalysts for Hydrogen Evolution
Source: Front Chem. 2020 Apr 7;8:170. doi: 10.3389/fchem.2020.00170 (PMC7154172; doi:10.3389/fchem.2020.00170)
Supplement: Supplementary file 1 [file Data_Sheet_1.PDF]

In situ Engineering of Hollow Porous Mo_2C@C Nanoballs Derived from Giant Mo-polydopamine Clusters as Highly Efficient Electrocatalysts for Hydrogen Evolution

Suli Liu,[†a,1] Xueqin Mu,[†a,1] Ruilin Cheng, [†b,1] Shiyu Lin,[a] Yang Zhu,[a] Changyun Chen,[*a] and Shichun Mu[*b]

[a]Department of Chemistry, Nanjing Xiaozhuang University, Nanjing, Jiangsu 211171, P. R. China.

[b]State Key Laboratory of Advanced Technology for Materials Synthesis and Processing, Wuhan University of Technology, Wuhan, Hubei 430056, P. R. China.

E-mail: msc@whut.edu.cn; yhjiangccy@126.com.

[1]These authors contributed equally to this work.

1 Experimental section

1.1 Synthesis of Mo-PD-3

250 mg ammonium molybdate tetrahydrate was added to 70 mL deionized water, the mixture was magnetically stirred at room temperature for 10 minutes, and then after adding 300 mg dopamine hydrochloride to the above solution, stirring was continued for another 40 minutes (In the same experimental procedure, 100 and 200 mg of dopamine hydrochloride were used to prepare comparative samples, which were respectively called Mo-PD-1 and Mo-PD-2). After the stirring was completed, 150 mL absolute ethanol was slowly poured into the above solution under constant stirring, and stirring was continued for 10 minutes, 0.4 mL ammonia was dropwise added to the precursor solution to maintain the pH of the solution to weakly alkaline during the 3 hours of stirring. After the reaction, A large amount of precipitate produced was separated by centrifugation and washed with deionized water and ethanol three times to remove the unreacted raw materials. Finally, the precipitate was

dried in a vacuum drying cabinet at 50° C for 12 hours to obtain Mo-PD-3.

1.2 Synthesis of hollow porous Mo_2C@C-X (X=1-3) nanoballs

The synthesized precursor Mo-PD-X (X=1-3) was transferred to a porcelain boat, and reacted at 800 °C for 5 hours at a heating speed of 5 °C/min in an Ar atmosphere.

1.3 Electrochemical measurements

Electrochemical experiments were performed on an Autolab electrochemical workstation (PGSTAT 302 N, Metrohm, Netherlands). For the measurementa a glassy carbon electrode (GCE) of 3 mm in diameter modified by synthesized material as the working electrode, graphite as counter electrode and the reference electrode needs to be used on a case-by-case basis. Ag/AgCl electrodes are used as reference electrodes in acidic and neutral electrolytes (0.5 M H_2SO_4 and 1.0 M PBS). Hg/HgO is used as reference in alkaline electrolyte (1.0 M KOH).

Before measurement, the bare GCE was mirror polished with alumina powder, followed by washing through ethanol and dried properly. Seperately, 3 mg of catalyst was properly suspended in a solution mixture having 20 µL Nafion solutions (0.5 wt%) and 1000 µL of a mixed solution of isopropanol and water(1:1), After processing the mixture in a cell pulverizer for 30 minutes, 5 µL dispersed catalyst ink was loaded on cleaned GCE and dried naturally, repeat the dropwise addition until the catalyst can form a flat and uniform catalyst layer on the electrode surface. As electrolyte 1 M KOH solution was used. Linear sweep voltammetry (LSV) at 5 mV/s scan rate and in the range of -0.8 V to -1.2 V was performed. As electrolyte 0.5 M H_2SO_4 solution was used. LSV at 5 mV/s scan rate and in the range of -0.1 V to 0.5 V was performed. The potentials in this work were calibrated to a reversible hydrogen electrode (RHE) scale according to the Nernst equation:

$$E_{RHE}=E_{Ag/AgCl}+0.059*pH+0.197\ V$$

$$E_{RHE}=E_{Hg/HgO}+0.059*pH+0.098\ V$$

1.4 Structural characterization

Phase characterization of the as-prepared materials was performed using powder X-ray diffraction (XRD, D8 ad, 2500VL/PC diffractometer equipped with Cu Kα radiation). Electron microscopic images of the synthesized catalysts were collected by

JEOL JSM 7100F Field Emission Scanning Electron Microscope (FE-SEM) were used and JEOL JEM 2100 Trans mission Electronic Microscope (TEM). ESCALAB 250 XPS System was utilized to record the X-ray photoelectron spectroscopy (XPS).

2 Supporting Tables

Table S1. HER catalytic data in alkaline environment.

Catalyst	Electrolyte	η_{10} (mV)	Tafel slope (mV dec^{-1})	Reference
Mo_2C@C-3	1.0 M KOH	115	61	This work
Pt/α-MoC_{1-x}	1.0 M KOH	140	78	*Adv. Funct. Mater.* **2019**, 29, 1901217.
Ni/β-Mo_2C	1.0 M KOH	157	61	*Chem. Commun.* **2018**, 54, 9901-9904.
MoO_2/MoC@C-8 50	1.0 M KOH	133	77.3	*Inorg. Chem. Front.* **2018**, 5, 446-453.
Ni/Mo_2C-PC	1.0 M KOH	179	101	*Chem. Sci.* **2017**, 8, 968-973.
Mo_2C@NC	1.0 M KOH	124	60	*Angew. Chem. Int. Ed.* **2015**, 54, 37.
MoS_2@N-doped carbon	1.0 M KOH	165	55	*Angew. Chem. Int. Ed.*, **2015**, 54, 7395.
Mo_2C-NC	1.0 M KOH	219.7	131	*ACS Appl. Mater. Interfaces* **2018**, 10, 42335−42347.
Mo_xC-Ni-carbon	1.0 M KOH	123	83	*Chem. Mater.* **2016**, 28, 6313-6320.
Mo_2C-Mo_3C_2	1.0 M KOH	116	64	*Nanoscale* **2019**, 11, 23318-23329.
α-MoC_{1-x}/NC	1.0 M KOH	118	84	*ACS Sustainable Chem. Eng.* **2019**, 7, 9637-9645.
CoMoO-S/NF	1.0 M KOH	134	87	*J. Catal.* **2018**, 361, 204-213.
Mo_2C-C	1.0 M KOH	149	66	*Nano Energy* **2017**,32,

				511-519.
MoC_x nano-octahedrons	1.0 M KOH	151	59	*Nat. Commun.* **2015**, 6, 6512.
α-Mo_2C nanoparticles	1.0 M KOH	176	58	*J. Mater. Chem. A* **2015**, 3, 8361-8368.

Table S2. HER catalytic data in acidic environment.

**Catalyst**	**Electrolyte**	**η_{10} (mV)**	**Tafel slope (mV dec^{-1})**	**Reference**
Mo_2C@C-3	0.5 M H_2SO_4	129	55	This work
α-MoC_{1-x}-MoP/ C	0.5 M H_2SO_4	173	57	*Electrochim. Acta* **2020**, 334, 135624.
N, P-Mo_2C@C	0.5 M H_2SO_4	141	56	*ACS Nano* **2016**, 10, 8851.
Zn-N-MoC-H NSs	0.5 M H_2SO_4	128	52.1	*Nanoscale* **2019**, 11, 1700-1709.
Mo_2C-carbon sphere	0.5 M H_2SO_4	164	85	*Nano Energy* **2017**, 32, 511.
α-MoC_{1-x}/NC	0.5 M H_2SO_4	142	74	*ACS Sustainable Chem. Eng.* **2019**, 7, 9637-9645.
L-Mo_2C	0.5 M H_2SO_4	170	77	*ACS Appl. Mater. Interfaces* **2018**, 10, 40500-40508.
Mo/α-MoC_{1-x}	0.5 M H_2SO_4	>120	-	*Adv. Mater. Interfaces* **2018**, 5, 1800223.
β-Mo_2C/C	0.5 M H_2SO_4	187	76	*ACS Sustainable Chem. Eng.* **2019**, 7, 9637-9645.
MoC_x octahedrons	0.5 M H_2SO_4	142	53	*Nat. Commun.* **2015**, 6, 6512.
Mo_2C-Mo_3C_2	0.5 M H_2SO_4	134	64	*Nanoscale* **2019**, 11, 23318-23329.
Mo_2C/VC@C	0.5 M H_2SO_4	122	43.8	*Nano Energy* **2019**, 60, 520-526.
Mo_2C-Ni-CMF0.2	0.5 M H_2SO_4	131	34.1	*J. Colloid Interface Sci.* **2019**, 543, 300-306.
0.56α-MoC_{1-x}/0.44β-	0.5 M H_2SO_4	155	48	*Appl. Catal. B: Environ.*

MoC				**2019**, 247, 78.
Co-Mo_2C-0.020	0.5 M H_2SO_4	140	40	*Adv.Funct.Mater.* **2016**, 26, 5590.
nanobelts-Mo_2C	0.5 M H_2SO_4	140	-	*Appl. Catal. B: Environ.* **2018**, 224, 533.
Mo_2C@GC-800-3.3	0.5 M H_2SO_4	125	66	*ACS Appl. Mater. Interfaces.* **2018,** 10, 18761-18770.
Mo_2C Nanocrystals on Mo foil	0.5 M H_2SO_4	255	100	*Adv. Sci.* **2018**, 5, 1700601.
2Hc-MoS_2 (250 ℃)	0.5 M H_2SO_4	191	64	*Adv. Mater.* **2017**, 29, 1703863.
